# No Benefit of Prophylactic Surgical Drainage in Combined Liver and Kidney Transplantation: Our Experience and Review of the Literature

**DOI:** 10.3389/fsurg.2021.690436

**Published:** 2021-07-12

**Authors:** Paolo Vincenzi, Jeffrey J. Gaynor, Linda J. Chen, Jose Figueiro, Mahmoud Morsi, Gennaro Selvaggi, Akin Tekin, Rodrigo Vianna, Gaetano Ciancio

**Affiliations:** ^1^Division of Kidney-Pancreas Transplantation, Department of Surgery, Miami Transplant Institute, Jackson Memorial Hospital, University of Miami Miller School of Medicine, Miami, FL, United States; ^2^Division of Liver and Intestinal Transplantation, Department of Surgery, Miami Transplant Institute, Jackson Memorial Hospital, University of Miami Miller School of Medicine, Miami, FL, United States; ^3^Department of Surgery, Miami Transplant Institute, University of Miami Miller School of Medicine, Miami, FL, United States; ^4^Department of Urology, Jackson Memorial Hospital, University of Miami Miller School of Medicine, Miami, FL, United States

**Keywords:** wound complications, DGF, prophylactic drainage, combined liver and kidney transplantation, graft failure

## Abstract

**Background:** Contrasting results have emerged from limited studies investigating the role of prophylactic surgical drainage in preventing wound morbidity after liver and kidney transplantation. This retrospective study analyzes the use of surgical drain and the incidence of wound complications in combined liver and kidney transplantation (CLKTx).

**Methods:** A total of 55 patients aged ≥18 years were divided into two groups: the drain group (D) (*n* = 35) and the drain-free group (DF) (*n* = 20). Discretion to place a drain was based exclusively on surgeon preference. All deceased donor kidneys were connected to the LifePort Renal Preservation Machine® prior to transplantation, in both simultaneous and delayed technique of implantation of the renal allograft. The primary outcome was the development of superficial/deep wound complications during the study follow-up. Secondary outcomes included the development of delayed graft function (DGF) of the transplanted kidney, primary non-function (PNF) and early allograft dysfunction (EAD) of the transplanted liver, graft failure, graft and patient survival, overall post-operative morbidity rate and length of hospital stay.

**Results:** With a median follow-up of 14.4 months after transplant, no difference in the incidence of superficial/deep wound complications, except for hematomas, in collections size, intervention rate, PNF, EAD, graft failure and patient survival, was observed between the 2 groups. Significantly lower level of platelets, higher INR values, DGF, morbidity rates and length of hospital stay were reported post-operatively in the D group. Pre-operative hypoalbuminemia and longer CIT were included in the propensity score for receiving a drain and were associated with a significantly higher rate of developing a hematoma post-transplant.

**Conclusions:** Absence of the surgical drain did not appear to adversely affect wound morbidity compared to the prophylactic use of drains in renal transplant patients during CLKTx.

## Introduction

Advances in surgical techniques and improvements in immunosuppression protocols have led to an appreciable reduction in post-operative morbidity and graft loss in solid organ transplantation (1, 2). Nevertheless, surgical complications are still considered to be a major cause of morbidity (3, 4).

Wound infections or dehiscence, incisional hernia, evisceration and fluid collections are the most common types of post-transplant surgical complications and are responsible for significant morbidity, prolonged hospitalization, high rates of hospital readmission, and increased costs (5–7). Though most of them are managed conservatively, others require interventions such as percutaneous drain insertion, vacuum-assisted closure, and surgery. Advanced age, diabetes, obesity, smoking history, poor nutritional status, specific immunosuppression protocols and peculiar surgical techniques are widely accepted risk factors for the development of these complications (6, 8, 9).

The reported incidence is highly variable, in the range of 4–27% for superficial and 0.6–51% for deep wound complications (8, 10). Most of the peri-graft collections are small, not associated with symptoms or graft dysfunction, and incidentally detected on routine ultrasound scanning (6, 11, 12). Although the peak incidence occurs at 2–6 weeks post-transplant, these complications have been known to develop at 6 months following surgery (8, 11–13).

It is common practice to place a surgical drain in the setting of abdominal organ transplantation with the aim to decompress the surgical site, prevent collections and monitor post-operative bleeding, bile or urine leakage (4, 5, 8). However, in most cases, these drains are removed days before the peak incidence of fluid accumulation.

Furthermore, previous research studies into the effectiveness of prophylactic drainage in reducing the morbidity rate following gastrointestinal (14), hepatic (15–17), vascular (18), thyroid (19), and breast surgery (20) have not universally substantiated a benefit to drainage.

While reports in the literature regarding the potential benefit of prophylactic surgical drainage are rather sporadic and show contrasting results as to whether there is any advantage of inserting drains after isolated liver and kidney transplantation (8, 21–29), there is currently no study that has addressed this topic in combined liver and kidney transplantation (CLKTx).

Therefore, the aim of this retrospective study was to evaluate the role of prophylactic drainage in potentially decreasing the incidence of wound-related complications after kidney transplantation in CLKTx.

## Materials and Methods

A retrospective cohort study was conducted on 55 adult patients undergoing CLKTx at the Miami Transplant Institute between January 1, 2017 and August 5, 2020. Follow-up was until September 30, 2020. Data were obtained from a prospectively maintained electronic database and complemented by review of clinical charts and donor data. The study was in accordance with our University of Miami Institutional Review Board and the Helsinki Declaration. Written informed consent was obtained from all subjects or a legal surrogate.

Based on intraoperative placement of a Jackson Pratt (JP) surgical drain during the kidney transplant, recipients were retrospectively divided into 2 groups: the control group with drain (D) and the study group drain-free (DF). Discretion to place a drain was based exclusively on surgeon preference, not on a case-by-case basis or on a specific patient risk factor model established for the development of wound complications. In the D group, drains were removed once output was <50 cc for 2 consecutive days.

The primary study endpoint was the incidence of a wound complication regarding exclusively the kidney transplantation, whether superficial (at or above the fascia) or deep (below the fascia). Superficial wound complications included subcutaneous seroma and hematoma, wound infection and wound dehiscence without fascia disruption, interesting the surgical incision of the kidney transplant, while deep complications included every type of collection occurred around the renal allograft during the study follow-up. Infection with a reported isolated microorganism was defined as a deep abscess regardless of the type of deep wound complication.

Wound infection was defined as an infection limited to skin or subcutaneous tissue, diagnosed within 30 days of operation with purulent drainage from the superficial incision or a sign or symptom of infection, such as pain, tenderness, heat, or swelling, with the incision left open, whereas wound dehiscence without fascia disruption was defined as a separation of the superficial layers in the absence of documented incisional infection (6).

Secondary study endpoints analyzed included the incidence of delayed graft function (DGF) of the renal allograft, primary non-function (PNF) and early allograft dysfunction (EAD) of the liver allograft, death-censored renal and hepatic allograft failure, death-uncensored renal and hepatic allograft loss, death with a functioning graft, overall post-operative morbidity rate and length of hospital stay.

DGF of the transplanted kidney was defined as the requirement of dialysis within the first post-operative week (30).

PNF of the liver allograft was defined as need for re-transplantation up to day 10 or death due to graft non-function (31), while EAD was determined by at least one of the following parameters: bilirubin ≥ 10 mg/dl on day 7; INR ≥ 1.6 on day 7 and AST or ALT > 2,000 IU/L up to day 7 (32).

Death-censored renal allograft failure was defined as the date of return to chronic dialysis or graft nephrectomy (33), while death-censored liver allograft failure as the need for re-transplantation or patient death within 1 year (34). Death-uncensored allograft loss included either allograft failure or death with a functioning graft.

Post-operative complications were classified according to Clavien and Dindo criteria (35) and overall morbidity was assessed with the Comprehensive Complication Index (CCI) (36).

All the variables analyzed are listed in Table 1.

**Table 1:** Variables analyzed.

**Baseline**	**Post-operative**
**Recipient variables:**- Demographics - BMI - MELD score (37) - History of Diabetes Mellitus - ESRD etiology - Timing of Kidney Failure (AKI vs. AKI on CKI vs. CKI) - Dialysis modality - Pre-transplant CVVH - Liver re-transplant - Kidney re-transplant - Pre-transplant Serum Albumin - Pre-transplant Platelet Count - Pre-transplant INR **Donor variables:** - Age - BMI - DCD status - KDPI (38) **Transplant variables:** - Timing KTx after LTx - CIT and WIT - Placement of ureteral stent	Lowest platelet count post-operative day 0–10 Highest INR value post-operative day 0–10 Overall incidence of wound complications Incidence and type of superficial wound complications Incidence and type of deep wound complications Maximum and Mean diameters of peri-renal collections Rate and type of intervention on peri-renal collections Incidence of DGF (renal allograft) Incidence of PNF and EAD (liver allograft) Incidence of death-censored renal allograft failure Incidence of death-censored hepatic allograft failure Incidence of death-uncensored renal graft loss Incidence of death-uncensored hepatic graft loss Incidence of death with a functioning graft Overall post-operative morbidity rate Length of hospital stay Bilirubin value at post-operative day 7 INR value at post-operative day 7 AST and ALT levels at post-operative day 7

*BMI, Body Mass Index; MELD, Model for End-stage Liver Disease; ESRD, End-Stage Renal Disease; AKI, Acute Kidney Injury; CKI, Chronic Kidney Injury; CVVH, Continuous Venous-Venous Hemofiltration; DCD, Donation after Cardiac Death; KDPI, Kidney Donor Profile Index; KTx, Kidney Transplant; LTx, Liver Transplant; CIT, Cold Ischemic Time; WIT, Warm Ischemic Time; DGF, Delayed Graft Function; PNF, Primary Non-Function; EAD, Early Allograft Dysfunction*.

### Surgical Technique

In our series, all deceased donor kidneys were connected to the LifePort Renal Preservation Machine® prior to transplantation, using kidney perfusion solution (KPS-1®), independently from adopting the simultaneous (at the time of liver transplant) or delayed (at a later time as a second operation) technique of implantation of the renal allograft.

Kidney transplant (KTx) was always performed using the standard extra-peritoneal approach. The right iliac fossa was the site for allograft implantation, except in one re-transplant case. The recipient's external iliac vessels were dissected free, with limited dissection to the anterior wall (not the whole circumference) of the external iliac vessels. After completing the vascular anastomoses, a modified extravesical ureteroneocystostomy technique was used in all recipients. Double J ureteral stent was placed in four cases.

All recipients received immunosuppressive therapy according to protocols at our center, with induction consisting of intravenous antithymocyte globulin (1 mg/kg × 3 doses), basiliximab (20 mg × 2 doses) and methylprednisolone (500 mg × 3 doses). First dose of each immunosuppressant drug was administered intraoperatively before reperfusion of renal allograft. Maintenance immunosuppression included a steroid-free regimen consisting of tacrolimus and mycophenolate mofetil, starting on post-operative day 1 (39).

Following surgery and prior to hospital discharge, a color-doppler and gray scale ultrasonography (US) was routinely performed in all recipients. In addition, US and/or abdominal CT scan were ordered at the discretion of the inpatient physician, based on clinical indications such as pain at the site of the graft, fever, hematuria, unexplained low or decreased urine output, anemia or elevated serum creatinine level.

### Statistical Analysis

Frequency distributions were determined for baseline categorical variables, and the mean along with standard error (±SE) were calculated for baseline continuous variables (with geometric mean and corresponding SE used instead of arithmetic mean for baseline continuous variables having skewed distributions). Tests of association between baseline variables and JP drain use (No/Yes) were performed using Pearson (uncorrected) chi-squared-tests and standard *t*-tests. Tests of association between JP drain use (No/Yes) and various outcome variables were performed using Pearson (uncorrected) chi-squared tests for dichotomous outcomes and log-rank tests for time-to-event outcomes. Multivariable analysis was performed using stepwise logistic and linear regression. *P*-values < 0.05 were considered to be statistically significant.

Stepwise logistic regression to determine the significant multivariable predictors of the likelihood of receiving a JP drain (yes/no) was specifically performed along with resulting propensity scores. Propensity scores are typically used as a way to control for the effects of any unbalanced distributions of other potentially important baseline prognosticators existing between two study groups (in this case, receiving vs. not receiving a JP drain).

## Results

In total, 35 patients were enrolled in the D group and 20 in the DF group; median follow-up was 14.4 (range: 1.1–35.6) months post-transplant. Overall, mean recipient age was 60.7 (±1.4) years, and 60% (33/55) of recipients were male.

Delayed implantation of the renal allograft was performed in 72.7% of cases (40/55), with a median time of 13.6 (range: 6.7–47.3) h after the liver transplant (LTx).

Tests of association of the baseline variables with JP drain use (No/Yes) found that the mean pre-operative serum albumin level was significantly lower in the D group (*p* = 0.006), while the mean pre-operative INR and cold ischemic time (CIT) were both significantly elevated in the D group (*p* = 0.04 and 0.01, respectively), as listed in Table 2. No significant differences were found among the other baseline parameters analyzed (Table 2).

**Table 2:** Baseline variables analyzed.

	**DF group** **(*n*: 20)**	**D group** **(*n*: 35)**	***P-*value**
**Recipient data**			
Age (years), mean ± SE	59.6 ± 2.2	61.4 ± 1.8	0.54
Male, *n* (%)	9 (45)	24 (68.6)	0.09
BMI (kg/m^2^), mean ± SE	26.3 ± 1.2	27 ± 0.8	0.63
**Race**, ***n*** **(%)**			
Afro-American	2 (10)	6 (17.1)	0.47
Hispanic	14 (70)	17 (48.6)	0.12
Caucasian	4 (20)	12 (34.3)	0.26
MELD score, mean ± SE	27.7 ± 1.4	29.2 ± 1.1	0.41
DM, *n* (%)	10 (50)	26 (74.3)	0.07
**ESRD etiology**, ***n*** **(%)**			
HRS	5 (25)	17 (48.6)	0.09
Metabolic (DM and/or HTN)	7 (35)	22 (62.9)	0.05
PKD	5 (25)	2 (5.7)	0.04
Glomerulonephritis	2 (10)	1 (2.9)	0.26
Other^a^	4 (20)	9 (25.7)	0.63
**Timing of kidney failure**, ***n*** **(%)**			
AKI	3 (15)	6 (17.1)	0.84
AKI on CKI	2 (10)	11 (31.4)	0.07
CKI	15 (75)	18 (51.4)	0.09
**Dialysis modality**			
Pre-emptive, *n* (%)	5 (25)	9 (25.7)	0.95
Hemodialysis, *n* (%)	15 (75)	26 (74.3)	0.95
Pre-transplant CVVH, *n* (%)	4 (20)	5 (14.3)	0.58
Liver re-transplant, *n* (%)	0 (0)	5 (14.3)	0.08
Kidney re-transplant, *n* (%)	0 (0)	1 (2.9)	0.45
Serum Albumin (g/dL), mean*/SE	3.69*/1.05	3.05*/1.04	0.006
Platelet count (per ml), mean*/SE	79.6*/1.2	55.7*/1.1	0.14
INR value, mean*/SE	1.36*/1.07	1.64*/1.06	0.04
**Donor data**			
Age (years), mean ± SE	36.4 ± 3.5	40.2 ± 2.4	0.36
BMI (kg/m^2^), mean ± SE	25.5 ± 1.1	25.5 ± 0.8	0.99
DCD, *n* (%)	4 (20)	6 (17.1)	0.79
KDPI (%), mean ± SE	41.6 ± 5.7	42.2 ± 3.9	0.93
**Kidney transplant data**			
Delayed KTx, *n* (%)	14 (35)	26 (65)	0.73
CIT (h), mean ± SE	18.5 ± 2.2	27.3 ± 2.2	0.01
WIT (min), mean ± SE	28.1 ± 1.6	30.8 ± 1.3	0.21
Placement of ureteral stent, *n* (%)	0 (0)	4 (11.4)	0.12

a *Calcineurin Inhibitor Toxicity, Focal Segmental Glomerulosclerosis, HCV or HIV nephropathy*.

*BMI, Body Mass Index; MELD, Model for End-stage Liver Disease; DM, Diabetes Mellitus; ESRD, End-Stage Renal Disease; HRS, Hepato-Renal Syndrome; HTN, Hypertension; PKD, Polycystic Kidney Disease; AKI, Acute Kidney Injury; CKI, Chronic Kidney Injury; CVVH, Continuous Venous-Venous Hemofiltration; DCD, Donation after Cardiac Death; KDPI, Kidney Donor Profile Index; CIT, Cold Ischemic Time; WIT, Warm Ischemic Time*.

In a stepwise logistic regression analysis of multivariable baseline predictors of JP drain use, 2 variables were selected containing independent predictive value: having a lower pre-operative serum albumin and higher CIT, (*p* = 0.01 and 0.02, respectively), with a propensity score for receiving a drain shown in Table 3. Dichotomizing the 2 selected multivariable predictors of JP drain use as pre-transplant serum albumin < vs. ≥3.4 g/dL and CIT as < vs. ≥24 h, the observed percentages of patients who received a JP Drain were as follows: 35.7% (5/14) vs. 44.4% (4/9) for CIT < vs. ≥ 24 h among patients with a normal serum albumin (≥3.4 g/dL); and 66.7% (10/15) vs. 94.1% (16/17) for CIT < vs. ≥24 h among patients with a low serum albumin (<3.4 g/dL).

**Table 3:** Selected logistic model (obtained *via* stepwise regression) for the likelihood of receiving a drain (35 events) and propensity score.

**Multivariable model variable**	***P-*value**	**Coeff ± SE**
Log [Pre-transplant Serum Alb (g/dL)]	0.01	−3.596 ± 1.506
CIT	0.02	0.063 ± 0.029
**Propensity score for receiving a drain:** 3.501 – [{3.596^*^ Log [Pre-transplant Serum Alb (g/dL)]} + (0.063^*^CIT)]		

*Alb, albumin; CIT, Cold Ischemic Time*.

Regarding the post-operative variables analyzed, the platelet count and INR value recorded in the first ten post-operative days differed between the two groups, with significantly higher degree of thrombocytopenia (*p* = 0.004) and coagulopathy (*p* = 0.04) observed in the control group (Table 4).

**Table 4:** Post-operative outcomes analyzed.

	**DF group** **(*n*: 20)**	**D group** **(*n*: 35)**	***P-*value**
Lowest PLT count (per ml) day 0–10, mean*/SE	33.65*/1.13	20.76*/1.1	0.004
Highest INR value day 0–10, mean*/SE	1.83*/1.06	2.16*/1.05	0.04
**Wound-related complications**, ***n*** **(%)**	11 (55)	22 (62.9)	0.57
Superficial, *n* (%)	2 (10)	5 (14.2)	0.64
Deep, *n* (%)	9 (45)	22 (62.9)	0.2
**Superficial wound complications**			
Subcutaneous Hematoma, *n* (%)	0 (0)	2 (5.7)	0.28
Wound dehiscence, *n* (%)	2 (10)	4 (11.4)	0.87
Wound infection, *n* (%)	0 (0)	1 (2.9)	0.45
**Deep wound complications**			
Hematoma, *n* (%)	4 (20)	18 (51.4)	0.02
Seroma, *n* (%)	4 (20)	2 (5.7)	0.1
Lymphocele, *n* (%)	1 (5)	1 (2.9)	0.68
Urinoma, *n* (%)	0 (0)	1 (2.9)	0.45
Abscess, *n* (%)	1 (5)	4 (11.4)	0.43
Any type of hematoma, *n* (%)	4 (20)	19 (54.3)	0.01
Max diameter peri-transplant collection (cm), mean ± SE	8.47 ± 1.31	8.32 ± 0.81	0.92
Mean diameter peri-transplant collection (cm), mean ± SE	6.77 ± 0.95	7.15 ± 0.72	0.77
**Intervention on peri-transplant collection**			
Any type^a^, *n* (%)	4 (44.4)	7 (31.8)	0.5
IR percutaneous drainage, *n* (%)	4 (100)	3 (42.9)	0.06
DGF, *n* (%)	2 (10)	14 (40)	0.02
PNF, *n* (%)	0 (0)	0 (0)	N/A
EAD, *n* (%)	0 (0)	1 (2.8)	0.44
Death-censored renal allograft failure, *n* (%)	1 (5)	7 (20)	0.12
Death-uncensored renal allograft loss, *n* (%)	1 (5)	8 (22.9)	0.09
Death-censored hepatic allograft failure, *n* (%)	0 (0)	1 (2.8)	0.44
Death-uncensored hepatic allograft loss, *n* (%)	0 (0)	2 (5.7)	0.27
Death with a functioning graft, *n* (%)	0 (0)	1 (2.9)	0.43
**Liver function indices at post-operative day 7**			
Bilirubin level (mg/dl), mean*/SE	1.5*/1.16	2.51*/1.14	0.02
INR value, mean*/SE	1.14*/1.03	1.32*/1.03	0.002
AST level (U/l), mean*/SE	46.32*/1.14	43.32*/1.10	0.69
ALT level (U/l), mean*/SE	111.40*/1.19	87.86/1.13	0.26
Length of hospital stay (days), mean*/SE	14.78*/1.19	33.08*/1.16	0.001
**Overall post-operative morbidity**			
Clavien-Dindo grade ≥ 3, *n* (%)	9 (45)	25 (71.4)	0.05
CCI, mean ± SE	36 ± 5.1	59.2 ± 4.6	0.002

a *Nephrostomy, surgical or IR drainage*.

*PLT, platelet; IR, Interventional Radiology; DGF, Delayed Graft Function; PNF, Primary Non-Function; EAD, Early Allograft Dysfunction; CCI, Comprehensive Complication Index*.

In general, wound-related complications were detected in 33 patients (60.0% of the entire cohort), with superficial and deep complications reported in 7 (12.7%) and 31 (56.4%) cases, respectively (Table 4).

Regarding superficial wound complications documented at the level of kidney transplant incision, wound dehiscence was the most common type observed, with an incidence of 10.0% (2/20) and 11.4% (4/35) in the drain-free and drain groups, respectively. Subcutaneous hematoma and wound infection were observed in 2 cases (5.7%) and in 1 case (2.9%) in the control group, respectively, while no patients experienced these complications in the study group. There were no significant differences in the occurrence of each category of superficial wound complications between the two groups, as shown in Table 4.

Regarding deep wound complications, hematoma was the most frequent peri-renal collection documented, with 20.0% (4/20) and 51.4% (18/35) incidences of occurrence in the DF and D groups, respectively, followed by seroma, reported in 20.0% (*n* = 4) vs. 5.7% (*n* = 2) in the DF and D groups, and abscess, 5.0% (*n* = 1) vs. 11.4% (*n* = 4) in the DF and D groups, respectively (Table 4). Both lymphocele and urinoma occurred in 2.9% (*n* = 1) of patients in the control group, whereas 5.0% (*n* = 1) and 0.0% (*n* = 0) of those included in the study group developed these complications, respectively. In relation specifically to the number of patients who developed any hematoma, above or below the fascia, the incidence of occurrence was 20.0% (4/20) and 54.3% (19/35) in the DF and D groups, respectively (Table 4).

In univariable analysis, exclusively the proportion of patients who developed either a deep hematoma or any type of hematoma was significantly higher in the drain group, (*p* = 0.02 and 0.01, respectively), although this association was not confirmed in multivariable analysis, as the propensity to receive a JP drain (i.e., patients with a lower pre-operative serum albumin and/or higher CIT) was more strongly associated with hematoma development than the actual use of a JP drain (Table 4). Dichotomizing the 2 predictors of propensity to receive a JP drain as pre-transplant serum albumin < vs. ≥3.4 g/dL and CIT as < vs. ≥24 h, the observed percentages of patients who developed any hematoma were as follows: 21.4% (3/14) vs. 33.3% (3/9) for CIT < vs. ≥24 h among patients with a normal serum albumin (≥3.4 g/dL); and 40.0% (6/15) vs. 64.7% (11/17) for CIT < vs. ≥24 h among patients with a low serum albumin (< 3.4 g/dL).

With regard to dimensions of peri-renal graft collections, maximum and mean diameters of these were similar in both groups, 8.47 ± 1.31 cm (*n* = 9) vs. 8.32 ± 0.81 cm (*n* = 22) and 6.77 ± 0.95 cm (*n* = 9) vs. 7.15 ± 0.72 cm (*n* = 22), respectively, as listed in Table 4.

Median time to development of superficial complications was 0.7 (range: 0.5–2.0) months; median time to development of deep complications was 5 days (range: 1 day−3 months) post-transplant.

Among the 31 patients with a peri-renal graft collection, in 4 (44.4%) and in 7 (31.8%) of the drain-free and drain groups, respectively, an intervention (percutaneous drain insertion, percutaneous nephrostomy or re-operation) was indicated. All of the 4 patients (100%, 4/4) in the DF group underwent percutaneous drainage vs. only 3 (42.9%, 3/7) in the D group. No significant difference was found in the intervention rate between the two groups (Table 4). At the time of any type of intervention, the JP drain placed during surgery was already removed.

Among 30/31 patients who developed a peri-renal transplant collection and had a resolution during the follow-up period, median time to resolution of the deep wound complications was 3.5 (range: 0.3–11.1) months post-transplant. Superficial wound complications were always managed conservatively.

In relation to the other endpoints, DGF showed a statistically significant difference (*p* = 0.02), occurring in 10.0% (2/20) and 40.0% (14/35) of patients in the study and control groups, respectively, while the incidences of PNF, EAD, death-censored renal and hepatic allograft failure, death-uncensored renal and hepatic allograft loss, death with a functioning graft did not significantly differ between the two groups, as listed in Table 4.

Regarding the parameters of liver function, bilirubin and INR levels at 7 days post-transplant were significantly higher in the D group (*p* = 0.02 and 0.002, respectively), while no difference was observed in mean transaminases levels (Table 4).

In regard to overall post-operative morbidity rate, the number of patients that developed a major complication classified as Clavien-Dindo grade ≥3 and the mean CCI were significantly increased in the D group (*p* = 0.05 and 0.002, respectively), as shown in Table 4. In addition, hospital stay was significantly longer in this group (*p* = 0.001).

However, these associations were not confirmed in multivariable analysis, as they were no longer statistically significant once the propensity to receive a JP drain was controlled (results not shown).

## Discussion

As far as we are aware, this is the first study to investigate the role of prophylactic drainage after KTx during CLKTx. Our study confirms that insertion of a drain might not prevent from developing wound-related complications.

The effect of drains on post-operative outcomes has been investigated extensively in the setting of various non-transplant surgical procedures (14–20). According to Gurusamy et al., the incidence of abdominal collections, either infected or requiring treatment after laparoscopic or open cholecystectomy, was not modified by the placement of a surgical drain (40). Fong et al. were the first to report that intraoperative drain insertion during elective liver resection did not lead to significant differences in hospital stay, morbidity, and mortality (16), while in a prospective randomized study in patients with chronic liver disease undergoing elective hepatic resection for hepato-carcinoma, prophylactic drainage was associated with increased rates of complications and infections (17).

However, due to peculiar factors existing among patients undergoing transplant surgery, such as immunosuppressive status, severe coagulopathy, thrombocytopenia and uremia, the abovementioned studies cannot be considered as a guide in the field of transplantation. On the other hand, only a modest number of studies have explored the relationships between prophylactic drainage and post-transplant complication rates, also with conflicting results.

In relation to isolated LTx, single institution studies reported no benefits of drain insertion in overall morbidity and mortality (21–24). However, the only systematic review reported on this theme in 2011 did not find any evidence to conclude whether routine abdominal drainage is useful or harmful in patients undergoing LTx (25).

In the setting of isolated KTx, divergent results are described in retrospective series on this topic. Indeed, while Derweesh et al. documented a significant reduction in peri-graft collections, deep venous thrombosis and lymphocele intervention rates when drains are used (45.2 vs. 16.0%, 14.3 vs. 4.9%, and 19.0 vs. 2.5%, respectively) (26), Sidebottom et al. found no significant difference in the occurrence of major and minor wound complications to support routine drain insertion (27).

For Cimen et al., the only advantage of prophylactic drainage during KTx relies in reducing the need for imaging leading to a potential reduction in costs (28). However, their study follow-up, extended to only 1 month after transplant, did not allow for the detection of most of the peri-graft collections that would be expected to occur.

A recent meta-analysis concluded that no robust practice recommendations can be made with respect to prophylactic drainage after KTx, given the significant limitations involved such as the non-randomized nature of the studies, the different lengths of follow-up mentioned, and the lack of clear definitions on imaging criteria and the proportion of patients undergoing imaging (8).

We could find no reports in the literature regarding the use of prophylactic drains during CLKTx. While at our institution, placement of a drain is routine practice during LTx; however, prophylactic drainage during KTx mostly depends on surgeon preference. For that reason, we decided to investigate the relation between intraoperative drain insertion during KTx and wound morbidity in patients undergoing CLKTx. Indeed, the overall sicker conditions of combined liver and kidney recipients, strictly related to the detrimental combined effects of end-stage liver and kidney disease, explain their higher morbidity and mortality, compared to isolated liver and kidney transplantation (41, 42). In addition, in this setting, severe coagulopathy and significant vasopressor requirements occurring after the LTx, justify our frequent approach of delayed implantation of the renal allograft, allowing stabilization of patient's hemodynamics and coagulopathy (41, 42).

Therefore, all of these points make our data difficult to compare with those existing in the literature on isolated liver and kidney transplantation.

As emerged in our study, major complications (peri-transplant collections) outnumbered the rates of minor or superficial wound complications. Indeed, the overall incidence of patients developing a peri-renal transplant collection in our cohort was 56.4% (31/55), specifically, 62.9% (22/35) in the drain group and 45.0% (9/20) in the non-drain group, higher than that reported in previous series [16 and 45.2% in Derweesh et al. (26), 1.9 and 3% in Sidebottom et al. (27), 19.8 and 26% in Cimen et al. (28), and 23.6 and 30.6% in Atray et al. (29)]. On the other hand, superficial wound complications rates were in line with what has been described in the relevant literature, in the range of 4–27% (8, 43). Extensive follow-up US imaging applied to each patient in our study that allows early detection of peri-renal graft collections, combined with a median follow-up of 14.4 months, might explain the increased occurrence of deep complications in our series.

Our results indicate that prophylactic placement of a surgical drain did not favorably or unfavorably affect wound morbidity. In particular, no significant difference was recorded for each single specific type of superficial and deep wound complications, except for the percentage of patients who developed a hematoma, both below and above the fascia, which was significantly increased in the drain group. However, this observed unfavorable effect of prophylactic drain placement was not confirmed in multivariable analysis, and more severe degree of thrombocytopenia and coagulopathy documented in the pre- and post-operative period in the drain group might explain the higher incidence of hematic collections reported rather than the drain itself.

Differently, the rate of lymphocele occurrence was quite low compared with the reported rates of 0.6–34% after renal transplantation (11), possibly secondary to our adopted technique of limited external iliac vessels dissection resulting in less disturbance of recipient lymphatics.

In addition, we analyzed the “intervention rate” as a measure of clinical significance and found that intraoperative insertion of a drain did not reduce the rates of clinically significant peri-graft collections, although we noticed an increasing trend in percutaneous drainage in the drain-free group. The limited data do not allow to suggest definitive conclusions on this theme and we cannot exclude that placement of a surgical drain might have a prophylactic role against re-intervention.

At the same time, no difference was observed in the maximum and mean dimensions of peri-graft collections in both groups.

In our series, patients with lower pre-operative albumin level and higher INR value or prolonged CIT were more likely to be assigned to the drain group. As pre-operative hypoalbuminemia is considered a risk factor for wound complications (44), our final logistic regression model identified lower serum albumin and higher CIT as significant determinants only for the development of a hematoma.

Regarding secondary outcomes, while incidence of death-censored renal and hepatic allograft failure, death with a functioning graft, death-uncensored allograft loss, PNF and EAD did not significantly differ between the two groups, DGF rates were significantly higher in the drain group, which could be directly related to increased CIT, a well-known risk factor for this complication (45, 46).

Similarly, we were able to identify a significant discrepancy in the overall post-operative morbidity rate between the two groups, with higher grade of generic complications reported in the drain group and consequent increased length of hospital stay. This may be related to the increased proportion of patients who developed DGF that alone accounts for a grade IV-a complication, according to the Clavien-Dindo classification.

At the same time, pre- and post-operative higher INR levels, hypoalbuminemia and prolonged CIT recorded in the drain group might contribute to worsen the baseline vulnerability of the population studied, rendering this class at greater risk of post-operative complications.

Whatever the reasons, these outcomes were also significantly associated with the propensity to receive a surgical drain and, once this propensity score was controlled in the multivariable model for one of these clinical endpoints, the test of association of drain use with that endpoint was no longer statistically significant, indicating that the relation between placement of a drain and greater likelihood of one or more of these poorer outcomes occurring was attributed to selection bias.

There are several limitations to this study. Although the data were prospectively collected, the analysis was retrospective in nature. In addition, the low frequency of some of the outcome variables analyzed, such as DGF, might be responsible for a low type II error. Another limitation was that no criteria were set for using vs. not using drain at the time of transplant in our patients, and the gold standard for comparing two groups such as D vs. DF would be to perform a randomized controlled trial.

In conclusion, absence of the surgical drain does not appear to adversely affect the rate of wound complications compared to prophylactic use of drains in renal transplant patients during CLKTx. The limited need-to-do dissection and improved surgical technique might be the keys for abandoning drain use even in the presence of higher risk factors for wound complication development.

## Data Availability Statement

The raw data supporting the conclusions of this article will be made available by the authors, without undue reservation.

## Ethics Statement

The studies involving human participants were reviewed and approved by University of Miami Institutional Review Board. The patients/participants provided their written informed consent to participate in this study.

## Author Contributions

PV and GC: study conception and design. PV: acquisition of data. PV and JG: analysis and interpretation of data. PV, JG, and GC: drafting of manuscript. LC, JF, MM, GS, AT, RV, and GC: critical revision of manuscript. All authors contributed to the article and approved the submitted version.

## Conflict of Interest

The authors declare that the research was conducted in the absence of any commercial or financial relationships that could be construed as a potential conflict of interest.
